# Stress hyperglycemia, cardiac glucotoxicity, and critically ill patient outcomes current clinical and pathophysiological evidence

**DOI:** 10.14814/phy2.14713

**Published:** 2021-01-19

**Authors:** Marc Scheen, Raphael Giraud, Karim Bendjelid

**Affiliations:** ^1^ Intensive Care Division University Hospitals Geneva Switzerland; ^2^ Geneva Hemodynamic Research Group Geneva Switzerland; ^3^ Faculty of Medicine Geneva Switzerland

**Keywords:** cardiogenic shock, glucotoxicity, heart, heart failure, insulin, stress hyperglycemia

## Abstract

Stress hyperglycemia is a transient increase in blood glucose during acute physiological stress in the absence of glucose homeostasis dysfunction. Its's presence has been described in critically ill patients who are subject to many physiological insults. In this regard, hyperglycemia and impaired glucose tolerance are also frequent in patients who are admitted to the intensive care unit for heart failure and cardiogenic shock. The hyperglycemia observed at the beginning of these cardiac disorders appears to be related to a variety of stress mechanisms. The release of major stress and steroid hormones, catecholamine overload, and glucagon all participate in generating a state of insulin resistance with increased hepatic glucose output and glycogen breakdown. In fact, the observed pathophysiological response, which appears to regulate a stress situation, is harmful because it induces mitochondrial impairment, oxidative stress‐related injury to cells, endothelial damage, and dysfunction of several cellular channels. Paradigms are now being challenged by growing evidence of a phenomenon called glucotoxicity, providing an explanation for the benefits of lowering glucose levels with insulin therapy in these patients. In the present review, the authors present the data published on cardiac glucotoxicity and discuss the benefits of lowering plasma glucose to improve heart function and to positively affect the course of critical illness.

## Graphical abstract

A review of the pathophysiological and clinical evidence surrounding the impact of stress hyperglycemia and cardiac glucotoxicity on cardiac physiology.

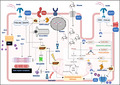

## 1 INTRODUCTION

Stress hyperglycemia describes a state of blood glucose deregulation that occurs during a period of acute physiological stress (Dungan et al., 2009; Marik & Bellomo, 2013). It is defined as an episode of hyperglycemia that resolves spontaneously after the dissipation of the acute illness (Dungan et al., 2009). Stress hyperglycemia is characteristically transient and is frequently observed in critically ill patients. Its presence has, however, been positively correlated with ICU mortality. Indeed, several studies have shown that controlling blood glucose fluctuations during catecholamine overload has survival benefits during acute illness such as acute myocardial infarction (AMI), stroke, and sepsis (Karetnikova et al., 2016; Lee et al., 2017; The NICE‐SUGAR Study Investigators, 2009; Van den Berghe et al., 2001, 2006). The same trends have been documented in a few prospective and retrospective observational studies in patients with cardiogenic shock (CS) (Abdin et al., 2018; Kataja et al., 2017; Lazzeri et al., 2015). Similar metabolic derangements also occur in patients with diabetes during acute illness, with surprising lower mortality than in controls without diabetes (Capes et al., 2000; Malmberg et al., 1995; Planer et al., 2013). It remains unclear why patients with diabetes seem to have survival benefits during an acute glucose insult in comparison to patients without diabetes. For several years, stress hyperglycemia was regarded as an epiphenomenon that reflected the severity of a critical illness (Marik & Bellomo, 2013). The extent to which hyperglycemia serves as a marker for more severe disease or simply predicts worse outcomes is still uncertain. Paradigms are now being challenged by growing evidence of a phenomenon called glucotoxicity (Dungan et al., 2009; Hirsch & O'Brien, 2012; Vanhorebeek et al., 2009; Vanhorebeek & Van den Berghe, 2006), providing an explanation for the benefits of lowering glucose levels with insulin therapy in these patients. It would seem that a new concept of cardiac glucotoxicity is now emerging, shedding light on the concept of stress hyperglycemia (Dungan et al., 2009; Hirsch & O'Brien, 2012; Marik & Bellomo, 2013; McCowen et al., 2001; Vanhorebeek et al., 2009; Vanhorebeek & Van den Berghe, 2006; Xiu et al., 2014). In the present review, the authors elaborate on data published on cardiac glucotoxicity among patients presenting with acute coronary syndrome, acute heart failure, and CS. The benefits of lowering plasma glucose to preserve heart function in these patients are then discussed.

### 1.1 Hyperglycemia in AMI

The first links between impaired glucose homeostasis and poor cardiac outcomes were first observed in patients with myocardial infarction (Capes et al., 2000; Cruickshank, 1931; Planer et al., 2013). Dysregulation of glycemia is extremely prevalent in acute coronary syndrome, with 70% of patients presenting dysglycemia (Arnold et al., 2014; Capes et al., 2000). Several studies show a direct correlation between blood glucose levels at admission and mortality in patients with AMI (Arnold et al., 2014; Kosiborod et al., 2005; Li et al., 2018; Malmberg et al., 1995; Timmer et al., 2011; Wahab Nazneem et al., 2002). The severity of stress hyperglycemia is directly linked to worse outcome in, as shown in the CardShock study with AMI patients complicated with CS (Kataja et al., 2017). The same trends are also observed in elderly patients with AMI as demonstrated by Kosiborod et al. (2005). Stress hyperglycemia in AMI is associated with an increased risk of in‐hospital mortality irrespective of the diabetes status (Capes et al., 2000).

Elevated glucose levels in the setting of AMI seems to predict the risk of complications (Leor et al., 1993). Leor et al. (1993) were one of the first groups to establish the association between hyperglycemia at admission and the development of CS. The same patients have an increased risk of congestive heart failure, and an increased incidence of reinfarction (Capes et al., 2000; Planer et al., 2013). In human studies on patients with myocardial infarction, hyperglycemia was associated with increased LV dysfunction, as well as more extensive myocardial necrosis, as reflected by the increased levels of cardiac enzyme markers and extensive microvascular obstruction on cardiac MRI (Chakrabarti et al., 2012; Jensen et al., 2011; Kosiborod et al., 2009).

The treatment of hyperglycemia in AMI patients is associated with survival benefits (Cheung et al., 2006; Kosiborod et al., 2009; Malmberg et al., 1995, 2005). Insulin therapy in AMI is associated with a 30% relative risk reduction in 1‐year mortality, as was demonstrated by Diabetes and Insulin‐Glucose infusion in Acute Myocardial Infarction (DIGAMI) trial (Malmberg et al., 1995). A retrospective analysis of 7820 hyperglycemic (admission glucose level >7.7 mmol/L) patients with AMI by Mikhail Kosiborod et al showed that glucose normalization after admission for AMI was correlated with better survival, regardless of whether the patient had received insulin (Kosiborod et al., 2009). Whether the mortality benefits solely stem from the lowering of blood glucose or whether insulin plays a role with its cardioprotective properties still needs to be elucidated. More high‐powered prospective trials are needed to fully comprehend the mechanisms at stake.

Patients with diabetes, previously treated with insulin, present attenuated manifestations of acute glucose fluctuations during AMI, when compared to patients having never received insulin (Capes et al., 2000; Malmberg et al., 1995; Planer et al., 2013). The reason for this phenomenon is still unclear (Capes et al., 2000; Malmberg et al., 1995; Planer et al., 2013). Concretely, hyperglycemia carries a worse prognosis in patients without diabetes and an acute ST‐elevation myocardial infarction (STEMI) than in patients with diabetes (Planer et al., 2013), possibly reflecting a form of protective preconditioning in the latter. This finding seems to distinguish the role and impact of an acute glucose elevation from that of chronic background hyperglycemia on prognosis (Capes et al., 2000; Lee et al., 2017; Malmberg et al., 1995; Planer et al., 2013). Analysis of the data provided by the CardShock trial also shows evidence pointing toward a protective effect of diabetes during hyperglycemia in CS (Kataja et al., 2017). Non‐surviving, patients without diabetes in the trial have significantly higher mean glucose levels than the non‐survivors with diabetes (Kataja et al., 2017). Several hypotheses have been elaborated to explain the survival benefit in patients with diabetes who are present with stress hyperglycemia. The first one is that patients with diabetes may have a higher likelihood of receiving insulin therapy in comparison to patients without the disease, possibly attenuating the rise in plasma free fatty acids (FFA) as well as providing antithrombotic properties associated with its use (Capes et al., 2000; Jain et al., 1993; Malmberg et al., 1999). Second, adaptive mechanisms by means of protective myocardial preconditioning to chronic hyperglycemia may come into play (Schaffer et al., 2000). Indeed, it was made evident by the work of Stephen W et al. that cardiomyocytes exposed in vitro to 72 h of background hyperglycemia, demonstrate increased expression of pro‐survival factor bcl‐2 as well as improved cytosolic calcium homeostasis (Schaffer et al., 2000). Chronic activation of protein kinase C (PKC) and its upregulation of pro‐survival pathways may also be involved (Schaffer et al., 2000).

The DIGAMI trial is a prospective randomized control trial involving coronary care units of 19 Swedish hospitals, that first established a mortality benefit of intensive blood glucose control with insulin on the outcome of patients with diabetes suffering AMI (Malmberg et al., 1995). Patients with an established diagnosis of diabetes or admission blood glucose levels of >11 mmol/L with or without a history of diabetes, and a diagnosis of MI in the previous 24 h were included in the trial and randomly assigned to receive insulin‐glucose infusion at admission followed by 3‐months insulin therapy or conventional therapy (Malmberg et al., 1995). The authors discovered a significant reduction in mortality at 1 year in the group that was assigned to insulin therapy, and distinguished newly diagnosed patients with diabetes from the ones with a prior history of the diagnosis (Malmberg et al., 1995). At 1 year, mortality rates in the insulin infusion group were 18.6% compared to 26.1% in the control group, corresponding to a relative mortality reduction of 29% (*p* = 0.027). The effect on mortality was even more pronounced in patients without a history of diabetes, and among patients with non‐insulin‐dependent diabetes who had never received insulin and carried a low‐risk cardiovascular profile (Malmberg et al., 1995). Since the mortality benefits were only significant in 1 year, it is not clear whether intervention during the peri‐infarct period or if long‐term glucose control that followed was the therapeutic intervention (Malmberg et al., 1995).

Curiously, the DIGAMI‐2 trial failed to live up to the excitement initially felt for the benefits of insulin administration in the AMI population (Malmberg et al., 2005). DIGAMI‐2 is a double‐blind, prospective RCT that randomized 1253 patients with myocardial infarction and an established type II diabetes to receiving either 24 h insulinglucose infusion followed by a subcutaneous insulin‐based long‐ term glucose control (Group 1), 24 h insulin–glucose infusion followed by standard glucose control (Group 2) or routine metabolic management according to local practice (Group 3). The primary endpoints were the short term and long‐term mortality among treatment groups 1 and 2. Secondary endpoints were a comparison of short and long‐term mortality between groups 2 and 3. Tertiary endpoints aimed at comparing differences among the study groups in the number of non‐fatal reinfarction, congestive heart failure, and stroke. DIGAMI‐2 failed to show mortality benefits of long‐term insulin administration in comparison to control groups for similar levels of glucose control (Malmberg et al., 2005). Mortality results plotted on Kaplan–Meier curves, after a follow‐up of 2 years, showed a result of 23.4% in group 1, 21.2% in group 2, and 17.9% in group 3 patients (Malmberg et al., 2005). Reasons why the trial failed may lie in the study design, where a few differences are notable between DIGAMI‐1 and DIGAMI‐2. First, the diabetes status and blood glucose levels of the patients included in both studies differed significantly. DIGAMI‐1 included all types of patients with an established diagnosis of diabetes as well as any patient, regardless of the previous diabetes status, with blood glucose levels of >11 mmol/L. DIGAMI‐2, moreover, focused on including only type II diabetes patients without any restrictions on the admission blood glucose as inclusion criteria. Patients in the DIGAMI‐2 trial, therefore, had better average glycemic controls compared to patients in DIGAMI‐1 (15.5±4.5 vs. 12.8±4.5 mmol/L), potentially explaining the difference in outcome between the two trials (Malmberg et al., 2005). Glucose lowering strategies with insulin resulted in more significant decreases in glycemia in DIGAMI‐1 (−5.8 mmol/L) than in DIGAMI 2 (−3.4 mmol/L), which was also reflected in the lowering of HbA1c (reduction by 1.4% in DIGAMI‐1 vs. reduction by 0.5% in DIGAMI‐2) (Malmberg et al., 2005).

Defining the value of stress hyperglycemia threshold in patients with diabetes is difficult and studies may have too low of a threshold to distinguish between elevations of glucose associated with baseline diabetes from elevations due to acute physiological stress (Capes et al., 2000; Malmberg et al., 1999). The baseline glycemia in the patients with diabetes included in most of these studies, in “unstressed” physiological state, is often not known (Capes et al., 2000; Kataja et al., 2017).

### 1.2 Hyperglycemia in CS

Hyperglycemia at admission is one of the strongest predictors of short‐term mortality in patients with CS (Abdin et al., 2018; Kataja et al., 2017; Leor et al., 1993; Tada et al., 2006). Peak glycemia is an independent predictor of mortality in STEMI patients that develop CS, and survivors show lower glycemic variability than nonsurvivors (Lazzeri et al., 2015). The retrospective analysis of subgroups in the Intra‐aortic Balloon Pump in Cardiogenic Shock II (IABP‐SHOCK II) confirmed the important prognostic role of impaired glycemic control on the survival of patients with CS (Abdin et al., 2018). A substudy of the IABP‐SHOCK II trial retrospectively analyzed the prognostic impact of glucose levels at admission in 513 patients with CS (Abdin et al., 2018). The results concluded that in patients with STEMI‐related CS, elevated glucose levels at admission are independent predictors of increased short‐ and long‐term mortality rates, in a manner that is independent of the diabetes status of the patients. Another finding from the Abdin et al substudy of the IABP‐SHOCK‐II trial was that STEMI patients with CS and increased glycemic values trended toward increased baseline lactatemia, had increased CPR requirements, had increased rates of mechanical ventilation, and increased short‐ and long‐term mortality rates independent of diabetes, stroke, or the type of shock (Abdin et al., 2018). Pöss et al. (2017) from the same group published a score called the IABP‐Shock II score that predicted mortality in patients who developed CS after STEMI. In this score, glucose levels at admission also emerged as one of the strongest predictors of short‐term mortality (Pöss et al., 2017). The score was published in 2017, with admission glucose levels >10.6 mmol/L being directly correlated with increased mortality (Abdin et al., 2018; Pöss et al., 2017). One of the limitations argued by the authors themselves was that the study was underpowered and involved only CS patients of ischemic origin. No prospective studies thus far have been carried out to investigate the impact of insulin and glycemic control on the incidence and mortality of CS patients.

## 2 PATHOPHYSIOLOGY OF CARDIAC GLUCOTOXICITY

### 2.1 Metabolism of glucose by cardiomyocytes

**FIGURE 1:**
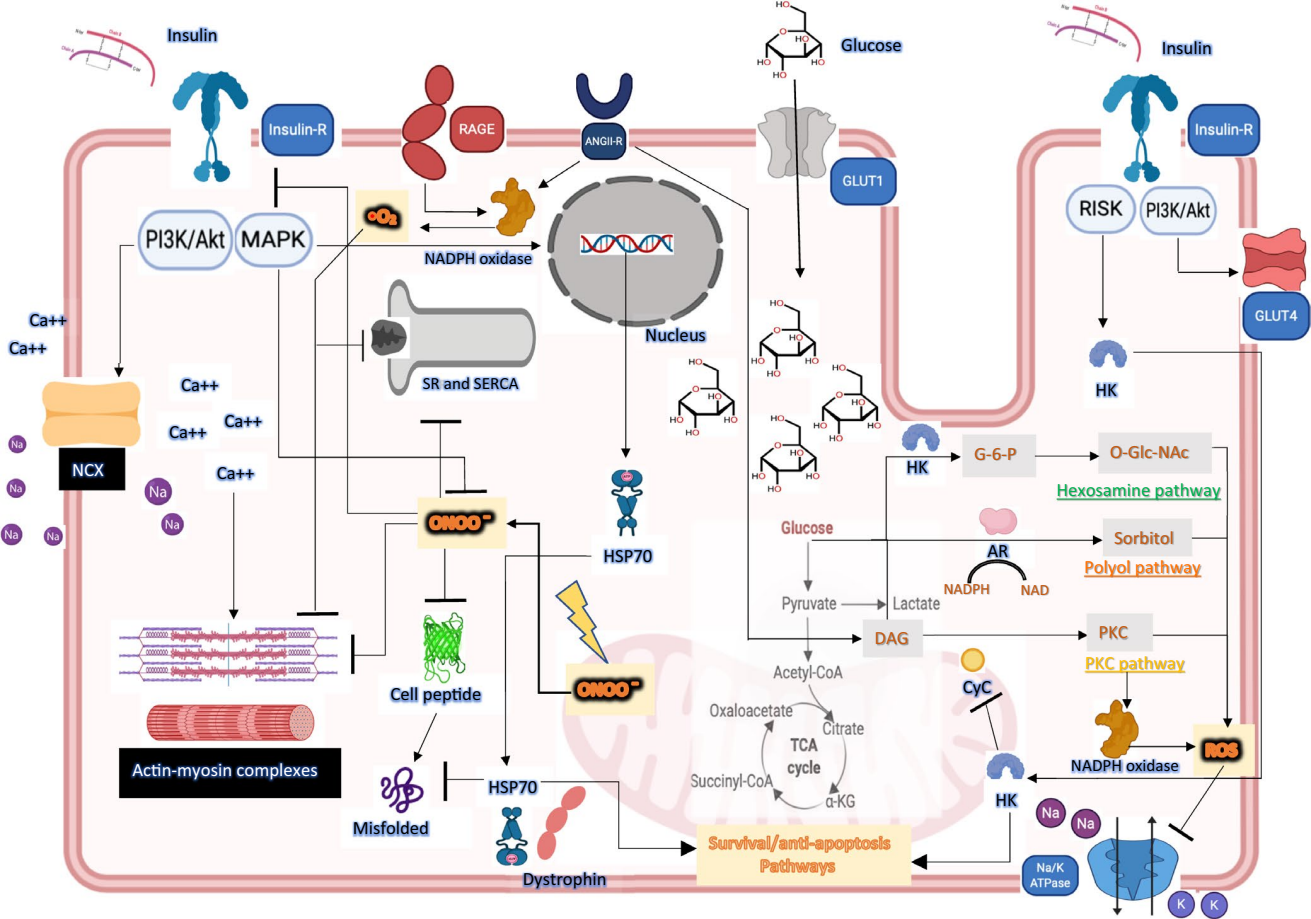
In states of acute physiological stress, glucose is shunted into cardiomyocytes via upregulated GLUT‐1. Glucose is metabolized via glycolytic and non‐glycolytic pathways, generating reactive oxygen species that target intracellular proteins that activate apoptotic pathways. The positive inotropic effects of insulin are mediated by the activation of the insulin receptor PI3 K/Akt pathway, leading to increased activity of NCX (a sodium‐calcium exchanger) and intracellular calcium accumulation. Insulin also counteracts the negative effects of peroxynitrite by inhibiting apoptotic pathways in cardiomyocytes. Part of this effect is mediated through the increased expression of HSP70 and its preferential localization to the plasma membrane with dystrophin. Insulin receptor activation also leads to the translocation of hexokinase (HK) to the mitochondrial membrane, where it exerts its cardioprotective effects via three mechanisms: (a) suppressing the generation of ROS, (b) inhibiting the release of cytochrome C (CyC) into the cytosol, and (c) inhibiting the formation of mitochondrial pores

During physiological aerobic fasting conditions, the heart predominantly consumes FFA, via the β‐oxidation pathway, in order to account for up to 60%–90% of cardiomyocyte ATP production (Figure 1) (Opie, 2014; Pascual & Coleman, 2016; Stanley et al., 2005). The remaining ATP production is accounted for by glycolysis and the oxidation of ketone bodies, lactate, and amino acids (Opie, 2014; Pascual & Coleman, 2016; Stanley et al., 2005; Szablewski, 2017). Glucose and lactate metabolism may account for up to 30% of myocardial ATP during basal state (Abel, 2004; Opie, 2014). Glucose enters the cardiomyocyte through specific glucose transmembrane transporters GLUT1 and GLUT4 (Shao & Tian, 2015; Szablewski, 2017). GLUT4 is an insulin‐dependent transporter responsible for the majority of glucose uptake in the adult heart in the postprandial state (Shao & Tian, 2015; Szablewski, 2017). It is predominantly present in intracellular vesicles at resting basal states, with its intracellular vesicles co‐containing 20% of total GLUT1 transporters (Kraegen et al., 1993). Its translocation to the plasma membrane may be induced by both insulin, and also catecholamines via α‐adrenergic stimulation (Rattigan et al., 1991; Szablewski, 2017). GLUT4 translocation is also coupled with myocardial contraction, with the conversion of AMP to ATP directly activating the AMPK pathway (Rattigan et al., 1991; Szablewski, 2017; Young et al., 2000). GLUT1 expression is substrate dependent, meaning that its expression increases during fasting and decreases in states of plethora, possibly a protective mechanism against glucose overload (Kraegen et al., 1993; Vanhorebeek & Van den Berghe, 2006). Reactive oxygen species (ROS) are generated as a result of normal aerobic cellular metabolism and are necessary for intracellular signaling in cardiomyocytes (Ide et al., 1999; Opie, 2014). They may also be generated as a byproduct of either xanthine oxidase activity, nitric oxide (NO) synthase uncoupling, activation of NADPH oxidases, cytochrome P450 pathway activation or through catecholamine autooxidation (Giordano, 2005). In physiological conditions, intracellular anti‐oxidant systems, buffer these ROS and prevent their accumulation and subsequent cellular injury from occurring (Conrad et al., 2004; Giordano, 2005; Kaul et al., 1993; Nordberg & Arner, 2001).

During states of metabolic imbalance such as during episodes of cellular hypoxia or ischemia, myocardial metabolism switches from aerobic mitochondrial FFA oxidation to cytosolic anaerobic glycolysis (Opie, 2014). This change is thought to be more energetically efficient, requiring lower oxygen consumption per unit ATP produced (Opie, 2014).

### 2.2 Pathophysiology of CS

CS has a 40–60% mortality rate (Hasdai et al., 1999; Hochman et al., 2000) and is the leading cause of death among patients with AMI (Goldberg et al., 1999; Leor et al., 1993). Although the principle diagnostic criteria are hemodynamic in nature, the metabolic and inflammatory changes that take place during tissue hypoperfusion are the major determinant of its prognosis (Abdin et al., 2018; Kataja et al., 2017). CS pathophysiology involves a markedly increased sympathetic nervous system tone and the release of major stress hormones that have a side effect of raising blood glucose levels. The secretion of hypothalamic, pituitary, and counterregulatory stress hormones, such as cortisol, adrenaline, noradrenaline, and glucagon, all participate in generating a state of insulin resistance with increased hepatic glucose output and glycogen breakdown (Dungan et al., 2009). The development of STEMI and its related CS are associated with a pro‐inflammatory state, accompanied by the release of proinflammatory cytokines and Damage‐associated molecular patterns (DAMP's) that alter patient prognosis and may potentiate insulin resistance (Anand et al., 2005; Latet et al., 2015; Pudil et al., 2001; Shpektor, 2010; Van Linthout & Tschöpe, 2017; Zhang et al., 2017). There seems to be growing evidence of both systemic and cardiac glucose‐mediated toxicity (Dungan et al., 2009; Guanghong et al., 2018; Guanghong et al., 2018; Hirsch & O'Brien, 2012; Isabella et al., 2019; Karina et al., 2014; Vanhorebeek et al., 2009; Vanhorebeek & Van den Berghe, 2006; Wold Loren et al., 2005) providing a link between the mortality benefits of lowering blood glucose concentrations and insulin therapy in “cardiac” patients.

### 2.3 The role of inflammation in myocardial injury

In both heart failure with preserved ejection fraction (HFpEF) and heart failure with reduced ejection fraction (HFrEF) patients, elevated levels of circulating inflammatory biomarkers are correlated with disease severity and increased mortality (Anand et al., 2005; Latet et al., 2015; Torre‐Amione et al., 1996; Van Linthout & Tschöpe, 2017; Zhang et al., 2017). CRP levels in HF patients are a marker of severity correlate directly correlated with mortality (Anand et al., 2005). Both innate and adaptive immune mechanisms are responsible for the adverse myocardial remodeling in HF patients that subsequentially leads to ventricular dysfunction (Blyszczuk et al., 2009; Seta et al., 1996; Sutton & Sharpe, 2000; Van Linthout & Tschöpe, 2017; Zhang et al., 2017). Elevated myocardial wall tension and stressed, necrotic cardiomyocytes release DAMPs that are potent activators of innate immunity (Liu et al., 2015; Zhang et al., 2017). These DAMPs, such as mitochondrial DNA, ATP and matrix proteins, activate the inflammatory cascade through the stimulation of Toll‐like receptors (mainly TLR4) and the NLRP3 inflammasome (Liu et al., 2015; Seta et al., 1996; Van Linthout & Tschöpe, 2017; Zhang et al., 2017). The resultant upregulation of NFκB induces the release of several inflammatory cytokines and chemokines (Anker & Coats, 2002; Liu et al., 2015; Zhang et al., 2017). IL‐1β, IL‐6, IL‐8, IL‐17, and TNF‐α are released by several immune cells in response to cardiomyocyte injury, leading to the local expression of soluble adhesion molecules (Liu et al., 2015; Seta et al., 1996; Van Linthout & Tschöpe, 2017; Zhang et al., 2017). A state of systemic inflammatory response syndrome with vasoplegia develop as tissue hypoperfusion persists, explaining why a significant proportion of CS nonsurvivors have a normal cardiac index but variable systemic vascular resistance profiles (Ferdinandy et al., 2000; Lim et al., 2003; Zhang et al., 2017). One potential explanation regarding this phenomenon seems to be the increased expression of inducible NO synthase (iNOs) by the vascular endothelium and cardiomyocytes (Feng et al., 2001; Ferdinandy et al., 2000; Förstermann et al., 1994; Lim et al., 2003). Excess NO is produced by iNOs under the influence of TNF‐α, IL‐1β, and IFN‐γ (Feng et al., 2001; Ferdinandy et al., 2000; Förstermann et al., 1994; Lim et al., 2003). NO at supraphysiological levels has negative inotropic effects and is responsible for systemic vasodilation (Feng et al., 2001; Ferdinandy et al., 2000; Förstermann et al., 1994; Lim et al., 2003). The heterogeneous profiles of peripheral vascular resistance are also explained by the secondary intestinal ischemia that follows the low‐flow state of patients with CS (Brunkhorst et al., 1999; Shpektor, 2010), with associated robust proteolysis (Bauzá‐Martinez et al., 2018). The increased permeability of the ischemic bowel favors bacterial and endotoxin translocation and increased systemic inflammation that further compromises prognosis (Brunkhorst et al., 1999; Shpektor, 2010).

At best there exists a statistical correlation linking glucose elevation to the degree of systemic inflammation in myocardial infarction (Marfella et al., 2003; Modan et al., 1975; Terlecki et al., 2013). Hyperglycemia leads to the elaboration of peroxynitrite that is responsible for cardiomyocyte apoptosis and cytokine‐induced decrease in myocardial contractility (Dungan et al., 2009; Ferdinandy et al., 2000; Marfella et al., 2009; Pacher et al., 2005). TNF‐⍺ is known to increase insulin resistance as was demonstrated by several human and animal studies through its action on insulin receptor signaling pathway (Dungan et al., 2009; Hotamisligil, 1999; Hotamisligil & Murray, 1994; Lang et al., 1992). In STEMI there is an associative link between elevated glycemia and inflammation as seen with increased leucocyte counts, increased CRP levels, and elevated IL‐18 levels (Marfella et al., 2003, 2009; Pacher et al., 2005; Terlecki et al., 2013). Furthermore, elevated blood glucose is responsible for exacerbating ischemia‐reperfusion injury by means of enhancing endothelin‐1 production by cardiomyocytes (Verma et al., 2002). Tight glycemic control during these ischemic insults has been associated with a reduction in early postinfarction remodeling (Marfella et al., 2009), pointing to a potential benefit of insulin administration in these situations.

### 2.4 Cardiac glucotoxicity, a refined concept

Cardiac glucose toxicity, which we will define as “Cardiac glucotoxicity,” provides a pathophysiological explanation for the observed negative outcomes on cardiovascular function. It reflects the variety of negative cardiometabolic effects observed secondary to a supraphysiological glucose insult (Brownlee, 2005; Cao et al., 2011; Mendelson Scott, 2008; Opie, 2014; Rossetti et al., 1990; Zeng et al., 2014). Indeed, glucotoxicity seems to affect myocardial function by altering both cardiomyocyte and endothelial cell function as made evident by human and animal models of diabetic cardiomyopathy. Human endothelial cells exposed to repetitive acute glucose fluctuations in vitro show signs of dysfunction with increased rates of apoptosis and elevated oxidative stress responses (Gunst & Schetz, 2009; Quagliaro et al., 2003).

From a molecular perspective, pathologic acute elevations in glycemia may result in the shunting of glucose via a noninsulin‐dependent GLUT‐1 transporter inside cardiomyocytes, as is the case in peripheral tissues (Dungan et al., 2009; Vanhorebeek & Van den Berghe, 2006). In a dog model of myocardial ischemia, GLUT‐1 mRNA and polypeptide expression were increased in ischemic cardiomyocytes (Feldhaus & Liedtke, 1998). GLUT‐1 upregulation has also been shown to occurs during critical illness, induced by cytokines and by the hypoxic‐ischemic environment through the possible upregulation of the hypoxia‐inducible factor‐1α in the heart (Abel, 2004; Brosius et al., 1997; Chen et al., 2001; Dungan et al., 2009; Feldhaus & Liedtke, 1998; Shao & Tian, 2015). In the diabetic cardiomyopathy model, glucose overload results in the accumulation of glycolysis substrates that carry several metabolic fates. These are the shunting via the polyol or the hexosamine pathway, cytosolic PKC activation or the generation of advanced glycation end products (AGE's) (Guanghong, Hill, et al., 2018; Guanghong, Whaley‐Connell, et al., 2018; Isabella et al., 2019; Karina et al., 2014; Shah & Brownlee, 2016; Sugamura & Keaney, 2011; Wold Loren et al., 2005). Dysregulated NO metabolism, as well as excessive RAAS and NADPH oxidase activity have been also implicated in the generation of cardiac ROS in the diabetic rat model (Guanghong, Hill, et al., 2018; Guanghong, Whaley‐Connell, et al., 2018; Isabella et al., 2019; Karina et al., 2014; Shah & Brownlee, 2016; Wold Loren et al., 2005).

### 2.5 The polyol pathway

The upregulation of the polyol pathway results in Aldose reductase mediated metabolism of glucose into sorbitol, consuming NADPH, and consequently depleting cytosolic glutathione levels (Bonnefont‐Rousselot et al., 2000; Chung et al., 2003; Guanghong, Hill, et al., 2018; Guanghong, Whaley‐Connell, et al., 2018; Isabella et al., 2019; Karina et al., 2014; Lee & Chung, 1999; Shah & Brownlee, 2016; Wold Loren et al., 2005). Polyol pathway activation has been linked to increased oxidative and osmotic stress in the rodent diabetic cardiomyopathy model (Bonnefont‐Rousselot et al., 2000; Chung et al., 2003; Guanghong, Hill, et al., 2018; Guanghong, Whaley‐Connell, et al., 2018; Isabella et al., 2019; Kador & Kinoshita, 1985; Karina et al., 2014; Lee & Chung, 1999; Liang‐jun, 2018; Shah & Brownlee, 2016; Wold Loren et al., 2005). Such mechanisms could be involved in the heart, during an acute supraphysiological glucose load, although little data are available. Wai Ho Tang et al did show a link between polyol mediated oxidative stress and Sarcoplasmic/Endoplasmic Reticulum Calcium ATPase (SERCA) mediated contractile dysfunction in the rat heart (Ho et al., 2010). His findings support oxidative stress‐mediated cardiac dysfunction attributed to polyol pathway activation (Ho et al., 2010). In this setting, glutathione depletion sets the grounds for creating a state of cellular imbalance between ROS and antioxidants (Bonnefont‐Rousselot et al., 2000; Chung et al., 2003; Guanghong, Hill, et al., 2018; Guanghong, Whaley‐Connell, et al., 2018; Ho et al., 2010; Isabella et al., 2019; Kador & Kinoshita, 1985; Karina et al., 2014; Lee & Chung, 1999; Liang‐jun, 2018; Shah & Brownlee, 2016; Wold Loren et al., 2005).

### 2.6 The hexosamine biosynthesis pathway (HBP)

Increased activation of the hexosamine pathway may also play a role in the negative cardiovascular outcomes observed in stress hyperglycemia (Guanghong, Whaley‐Connell, et al., 2018; Karina et al., 2014). Post‐translational alterations of SERCA and calcium/calmodulin‐dependent protein kinase result in altered calcium homeostasis and decreased diastolic relaxation in the rodent diabetic cardiomyopathy model (Guanghong, Hill, et al., 2018; Guanghong, Whaley‐Connell, et al., 2018; Karina et al., 2014; Yokoe et al., 2010). The elevated cytosolic generation of O‐linked N‐Acetylglucosamine (GlcNAc), a byproduct of the hexosamine pathway, is implicated in post‐translational modifications of phospholamban that directly results in SERCA inhibition leading to diastolic dysfunction (Guanghong, Hill, et al., 2018; Guanghong, Whaley‐Connell, et al., 2018; Karina et al., 2014; Yokoe et al., 2010). O‐linked GlcNAc, modifications have also been observed on actin filaments, explaining in part the decreased submaximal force observed in diabetic hearts (Ho et al., 2010). Basal short term activation of this pathway has been shown to be cardioprotective and promote survival pathways (Jones et al., 2008). More chronic activation of the HBP, for the timing of several weeks, seems to be what is deleterious for the cardiomyocyte, and therefore put in doubt its involvement in the negative cardiovascular prognosis in the setting of acute stress hyperglycemia (Guanghong, Hill, et al., 2018; Guanghong, Whaley‐Connell, et al., 2018; Karina et al., 2014; Ramirez‐Correa et al., 2008; Yokoe et al., 2010).

### 2.7 Hyperglycemia and oxidative stress

NADPH oxidase activation is the main source of ROS in the heart, forming superoxide radicals through the consumption of NADPH and molecular oxygen (Karina et al., 2014; Sugamura & Keaney, 2011). The two other main sources are the mitochondrial respiratory chain that generates superoxides and the NO synthase (Brand et al., 2004; Karina et al., 2014; Nishikawa et al., 2000; Sugamura & Keaney, 2011). NADPH oxidase has been involved in the pathogenesis of diabetic cardiomyopathy, with evidence principally obtained from the animal model (Pagano et al., 1998). It may be activated by means of angiotensin II (ANG II), endothelin‐1 (ET‐1), RAGE receptor signaling, and TNF‐α (De Keulenaer et al., 1998; Karina et al., 2014; Laskowski et al., 2006; Li et al., 1999; Moller, 2000; Nakagami et al., 2003; Pagano et al., 1998). Its activity is increased in diabetic hearts, causing increased oxidative stress and promoting LV interstitial fibrosis with cardiomyocyte hypertrophy (Karina et al., 2014; Li et al., 2002; Ritchie et al., 2005; Ritchie & Delbridge, 2006). Increased expression of NFkB, with the expression of profibrotic genes and matrix metalloproteinases activation seem to mediate the pro‐fibrotic changes observed (Karina et al., 2014; Seddon et al., 2007). In the acute hyperglycemia mouse model, there is evidence that an acute elevation of glucose leads to an increase in infarct size during reperfusion in AMI, mediated increased NADPH oxidase activity (Zequan et al., 2009).

ROS are also generated by the metabolism of glucose via its “alternative metabolic pathways” in the heart, being the polyol pathway and the hexosamine biosynthesis (Karina et al., 2014; Leor et al., 1993; Torre‐Amione et al., 1996). The end result is a state of dysregulated calcium homeostasis that seems to also contribute to both the systolic and diastolic dysfunction observed in the diabetic cardiomyopathy (Karina et al., 2014; Leor et al., 1993; Talukder et al., 2008). Indeed, calcium reuptake by the sarcoplasmic reticulum is reduced, slowing diastolic relaxation (Guanghong, Hill, et al., 2018; Leor et al., 1993; Talukder et al., 2008). Although evidence from animal studies seems to point toward the pathophysiological implication of the cited pathways in the chronic cardiac dysfunction of patients with diabetes, more evidence on their role during stress hyperglycemia is needed.

### 2.8 Role of AGEs in diabetic cardiomyocytes

Advanced glycation end products (AGEs) have been implicated in the pathogenesis of chronic complications of diabetes and aging (Candido et al., 2003; Jia et al., 2016; Jyotiska et al., 2018; Karina et al., 2014; Leor et al., 1993; Zequan et al., 2009). Their participation in cardiac pathology during diabetes has also been demonstrated in the rodent model, possibly acting either by isolated cellular protein cross‐linking or by means of the RAGE receptor signaling (Jyotiska et al., 2018; Karina et al., 2014; Leor et al., 1993). Modifications in extracellular matrix proteins such as collagen have been observed as a consequence of AGE pathobiology (Jyotiska et al., 2018; Karina et al., 2014; Leor et al., 1993). Although AGE mediated pathology is regarded as a chronic process, with gradual build‐up taking up to decades to be observed in humans, there is evidence pointing toward its implication in the acute setting (Jyotiska et al., 2018; Karina et al., 2014; LaPar et al., 2012; Leor et al., 1993; Zequan et al., 2009). Indeed, a study published in the American journal of respiratory cell and molecular biology in 2011, demonstrated that acute hyperglycemia mediated ischemia of reperfusion in the post‐transplant rodent lung model involved RAGE receptor activation (LaPar et al., 2012). RAGE receptor knock out attenuates ischemia of reperfusion during an acute glucose insult (LaPar et al., 2012). Indeed, RAGE receptor activation directly stimulates the activity of NADPH oxidase, potentiating the formation of ROS (Jyotiska et al., 2018; Karina et al., 2014; Leor et al., 1993).

### 2.9 PKC pathway activation

In diabetic cardiomyocytes, glucose overload leads to diacylglycerol accumulation, which is responsible for the activation of β and δ isoforms of PKC (Davidoff et al., 2004; Falcao‐Pires et al., 2011; Geraldes & King, 2010; Karina et al., 2014; Leor et al., 1993). PKC is directly responsible for myocardial ROS generation through the activation of NADPH oxidase (Karina et al., 2014; Leor et al., 1993; Zeng et al., 2014). PKC activation in the diabetic heart induces cardiomyocyte hypertrophy and apoptosis and increases the expression of connective tissue growth factor and transforming growth factor‐β1 (Davidoff et al., 2004; Falcao‐Pires et al., 2011; Geraldes & King, 2010; Karina et al., 2014; Koya et al., 1997; Leor et al., 1993; Wakasaki et al., 1997). The latter have been linked to the systolic and diastolic dysfunction observed in hearts of patients with diabetes (Karina et al., 2014; Wakasaki et al., 1997). Other molecular targets of activated PKC include cytosolic phospholipase A2, the Na/K‐ATPase, and transcription factors of various profibrotic, procoagulant (PAI), and pro‐inflammatory genes (TNF‐α and NFkB), linking glucose overload to inflammation, oxidative stress, and myocardial fibrosis (Karina et al., 2014; Koya et al., 1997; Leor et al., 1993). Once again, the role of PKC activation in cardiomyocytes during acute stress hyperglycemia is unknown.

### 2.10 Altered FFA metabolism

During states of systemic insulin resistance, the resultant decreased GLUT4 translocation at the plasma membrane contributes to the decreased availability of glycolytic substrates for cardiomyocytes, shifting the metabolic pathway to the excessive consumption of FFA (Chakrabarti et al., 2012). Fatty acid metabolism is well known to necessitate more oxygen consumption in comparison with that of glycolysis. The excessive FFA oxidation during ischemia is believed to contribute to decreased myocardial contractility and increases the risk of arrhythmia (Chakrabarti et al., 2012). In states of reduced oxygen delivery and increased myocardial oxygen demand, the inability to switch to glucose oxidation increases myocardial susceptibility to LV dysfunction and ischemia (Liu et al., 1996; Lopaschuk Gary & Stanley, 1997).

### 2.11 Endothelial NO synthase and its role in cardiac function

From a physiological perspective, insulin influences the bioavailability of cardiomyocyte NO by stimulating the activity of the eNO synthase inside endothelial cells (Jia et al., 2016; Muniyappa et al., 2007; Nathan & Xie, 1994; Zeng & Quon, 1996). The deficit in NO that results from the insulin resistance state negatively affects both the coronary circulation and the myocardial contractile apparatus (Karina et al., 2014; Leor et al., 1993; Muniyappa et al., 2007). Decreased NO bioavailability leads to decreased coronary vasodilatory reserve and diastolic dysfunction in cardiomyocytes (Jia et al., 2016; Karina et al., 2014; Leor et al., 1993; Muniyappa et al., 2007). The latter occurs by means of calcium cytosolic overload from decreased SERCA activity as well as through the phosphorylation of titin into a stiff non‐compliant isoform (Jia et al., 2016; Karina et al., 2014; Leor et al., 1993).

### 2.12 Contractile proteins and glucose overload

Hearts of patients with diabetes demonstrate alterations in contractile protein expression and phosphorylation, both linked to the development of LV systolic and diastolic dysfunction (Jia et al., 2016). Chronic hyperglycemia results in a shift in myosin heavy chain expression from a V1 to a V3 isoform (Cai & Kang, 2001; Guanghong, Hill, et al., 2018; Pollack et al., 1986). The phosphorylation of troponin I also contributes to the contractile dysfunction that accompanies diabetic cardiomyopathy, by altering myosin light chain‐2 and troponin I cross‐talk (Guanghong, Hill, et al., 2018; Pollack et al., 1986). Several cardiomyocyte hypertrophy related genes, such as β‐myosin heavy chain, insulin‐like growth factor 1 receptor, and B‐type natriuretic peptide are upregulated in the diabetic cardiomyopathy by means of increased oxidative stress and insulin resistance (Cai & Kang, 2001; Guanghong, Hill, et al., 2018; Jia et al., 2016; Karina et al., 2014; Leor et al., 1993).

### 2.13 Procoagulant effect of hyperglycemia

The procoagulant effect of hyperglycemia may provide another link in the explanation of the increased mortality observed in AMI and CS patients. Hyperglycemia mediates the transcription of coagulation factors such as tissue factor, Factors VII and VIII, induces direct platelet activation, and exposes coagulation factors through the disruption of the endothelial glycocalyx layer (Boden et al., 2007; Ceriello, 1993; Ceriello et al., 1988; Lemkes et al., 2010; Rao et al., 1999). Non‐enzymatic glycation of coagulation factors has also been implicated in its procoagulant pathogenesis (Lemkes et al., 2010). Acute hyperglycemia in patients without diabetes increases the procoagulant activity of Factor VII and increases thrombin–antithrombin complexes and soluble tissue factor (sTF) in an insulin‐independent manner (Lemkes et al., 2010; Stegenga et al., 2008). The hyperinsulinemia that accompanies acute hyperglycemia has been shown to increase levels of PAI‐1, thus leading to the inhibition of fibrinolysis and further increasing prothrombotic activity (Lemkes et al., 2010; Stegenga et al., 2008). It would seem that hyperglycemia and hyperinsulinemia are both independently responsible for the development of a prothrombotic state, each having the ability to potentiate the another. Evidence of the prothrombotic activity of glucose is also observed in patients with acute MI. Admission glucose >7.0 mmol/L in patients with STEMI has been linked to significantly increased thrombin‐antithrombin complexes and platelet activation, as measured by soluble CD40 ligand levels (Lemkes et al., 2010; Vaidyula et al., 2006).

## 3 THE EFFECTS OF INSULIN

### 3.1 Insulin and its cardioprotective effects

**FIGURE 2:**
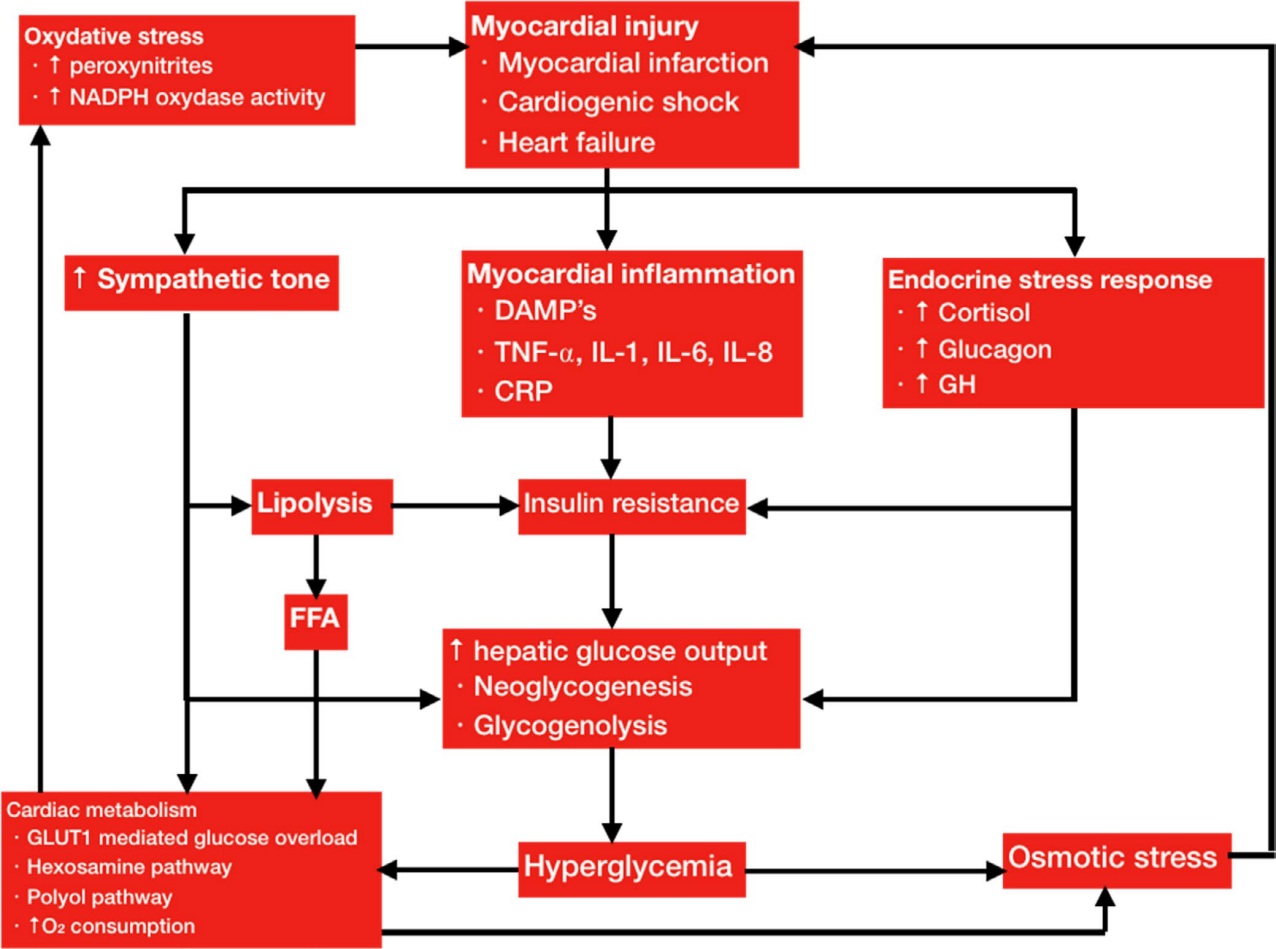
Pathophysiology of stress hyperglycemia and cardiac glucotoxicity

Insulin exerts positive cardiovascular effects by directly altering cardiomyocyte cellular biology (Figure 2) (Dandona et al., 2009; Ng et al., 2012). Insulin administration seems to counteract the negative systemic effects of glucotoxicity, while exerting positive inotropic effects on the myocardium (Dandona et al., 2009; Hsu et al., 2006; Ng et al., 2012). The ability to counteract oxidative stress‐mediated cellular injury by suppressing peroxynitrite (ONOO^−^) production, as well as its ability to inhibit cardiomyocyte apoptosis, make insulin an interesting potential therapeutic strategy for conditions such as acute heart failure and CS (Ji et al., 2010; Kline et al., 1997; Ng et al., 2012). The positive inotropic effects increase with insulin dose and are mediated by both calcium‐dependent and calcium‐independent mechanisms (Hsu et al., 2006; Lewinski et al., 2005; Lucchesi et al., 1972). Von Lewinski and collaborators showed that insulin transiently increases intracellular calcium levels by means of enhancing the activity of the sodium‐calcium exchanger on the sarcolemma of cardiomyocytes and, consequently, increasing sarcoplasmic reticulum calcium content via enhanced activity of SR calcium‐ATPase (Lewinski et al., 2005; Maier et al., 1999; Muniyappa et al., 2007). These positive inotropic effects are independent of both ß‐adrenergic stimulation and the rate of myocardial glycolysis (Lee & Downing, 1976; Lucchesi et al., 1972; Maier et al., 1999). Insulin receptor activation also leads to increased cardiomyocyte expression of HSP70, a chaperone protein. Insulin treatment administered in rats after 30 min of ischemia leads to the preferential colocalization of Heat Shock Protein 70 (HSP70) to the plasma membrane with dystrophin (Li et al., 2006). This phenomenon has been correlated with improved myocardial recovery during ischemia‐reperfusion injury (Li et al., 2006). It is thought that the myocardial protective effects of HSP70 localization are due to the activation of prosurvival cell signaling pathways when HSP70 colocalizes with dystrophin at the plasma membrane (Li et al., 2006). Reperfusion injury salvage kinase pathway signaling (RISK signaling), initiated by insulin receptor activation, leads to the translocation of intracellular hexokinase to the mitochondrial membrane, which is an event that leads to the inhibition of cardiomyocyte apoptosis (Da‐Silva et al., 2004; Ng et al., 2012; Pastorino et al., 2002). Hexokinase translocation to the mitochondrial membrane suppresses the generation of ROS by the hexosamine pathway and inhibits the release of cytochrome C into the cytosol, preventing the activation of caspases and the activation of proapoptotic pathways (Lewinski et al., 2005; Lucchesi et al., 1972; Marfella et al., 2003). Furthermore, hexokinase translocation inhibits the opening of mitochondrial transitional pores, thus preventing the permeabilization of mitochondrial membranes (Da‐Silva et al., 2004; Ng et al., 2012; Pastorino et al., 2002). Insulin seems to exert its cardioprotective effects via alterations in vascular tone. Indeed, insulin binding to its receptor activates the PI3 K‐Akt pathway, which is responsible for the induction of NO synthase in endothelial cells (Brownsey et al., 1997; Ng et al., 2012). The increased NO production allows for coronary vasodilation and increased coronary blood flow, as well as vasodilation in skeletal muscle vasculature (Ng et al., 2012; Rogers et al., 1977). Widespread decreased peripheral vasculature resistance, and redistribution of blood flow may favor cardiac output increase (Aljada & Dandona, 2000; Ng et al., 2012).

### 3.2 Anti‐inflammatory effects of insulin

The anti‐inflammatory properties of insulin are mainly attributed to its ability to reduce oxidative stress (Dandona et al., 2009; Ng et al., 2012). However, direct suppression of several immune checkpoints is observed with insulin infusion. Insulin administration during acute STEMI was associated with decreased levels of CRP of up to 40% and decreased serum amyloid protein during the initial 24‐h period (Chaudhuri et al., 2004; Dandona et al., 2009; Wong et al., 2004). From a molecular standpoint, insulin inhibits key intracellular signaling pathways, such as the MAPK pathway, JNK, and NFκB, resulting in decreased plasma levels of IL‐6, TNF‐ α and endothelial adhesion molecules (Dandona et al., 2009; Ng et al., 2012).

### 3.3 Clinical evidence of the effect of insulin on cardiovascular endpoints

Randomized control trials evaluating the effects of insulin on the incidence of chronic heart failure in patients with diabetes, failed to show any benefits of its administration in lowering the hospitalization rates for heart failure (Fitchett David et al., 2017; Gilbert & Krum, 2015; Pocock et al., 2006; The ORIGIN Trial Investigators, 2012). No large scale randomized prospective data are currently available concerning the effects of insulin on clinical outcomes in patients with established heart failure (Fitchett David et al., 2017; Gilbert & Krum, 2015). In fact several papers exist on the matter but none have been designed to or contained adequate power in order to analyze the correlation between insulin and the incidence of heart failure (Fitchett David et al., 2017; Gilbert & Krum, 2015). The retrospective evidence available is more controversial, with studies showing either no mortality differences in comparison to HF patients with diabetes treated with oral antidiabetic agents, or increased mortality in insulin‐requiring patients (Fitchett David et al., 2017; Gilbert & Krum, 2015). Part of the explanation for the latter could be the increased severity of disease that accompanies insulin‐requiring diabetes.

The BARI‐2D trial is a prospective RCT that included 2368 patients with type II diabetes and stable coronary artery disease to receive either prompt revascularization (percutaneous coronary intervention or coronary‐artery bypass grafting) with intensive medical therapy or intensive medical therapy alone followed by insulin‐sensitization or insulin‐provision therapy alone (Frye et al., 2009). The primary outcome measured was the rate of death of any cause at 5 years. The secondary endpoint measured was a composite of death and major cardiovascular events, being myocardial infarction and stroke. After a follow‐up of 5 years, there were no statistically significant differences for both primary and secondary outcomes between the chosen revascularization versus medical strategy and between the insulin‐sensitizing versus insulin‐provision group (Frye et al., 2009). The 5‐year rate of survival rate in the revascularization group was 88.3% with 87.8% survival rate in the medical therapy group. When comparing the insulin strategies, the 5‐year survival rate in the insulin sensitization group was 88.2% I comparison to 87.9% in the insulin­ provision group (Frye et al., 2009). There were no significant differences in the rate of freedom from major cardiovascular events among the compared groups (Frye et al., 2009).

The clinical evidence of insulin administration on outcomes in myocardial infarction remains mixed and inconclusive. The CREAT‐ECLA trial disproved what earlier trials on the benefits of glucose‐insulin and potassium (GIK) infusion in myocardial infarction showed (Fitchett David et al., 2017; Gilbert & Krum, 2015; Mamas Mamas et al., 2010; The CREATE‐ECLA Trial Group Investigators*, 2005). The early administration of GIK in the pre‐hospital setting during AMI still needs further research and no conclusions to this day may be withdrawn. One of the latest meta‐analyses published by Mamas et al. (2010) failed to show a mortality benefit of GIK in AMI, despite the data collected from 16 randomized trials with 28,000 patients. Both DIGAMI and HI‐5 studies did not show early mortality benefits of insulin administration in AMI (Cheung et al., 2006; Malmberg et al., 1995). Both trials do, however, point toward a benefit of good glycemic controls on AMI outcome, although solely the 1 year‐mortality of the DIGAMI trial was significantly lower for the insulin‐treated group (Cheung et al., 2006; Malmberg et al., 1995). A major critique of the HI‐5 study is the mean duration of symptom onset of 13 h to the initiation of insulin therapy (Cheung et al., 2006). The DIGAMI trial and the Dutch GIK trial also included patients up to 24 h after onset of symptoms (Malmberg et al., 1995; Van der Horst et al., 2003). Patients included in the intervention group of the DIGAMI trial also benefited from an additional 3 months of insulin therapy which could potentially account for the long‐term mortality benefit observed at 1 year (Malmberg et al., 1995). We, therefore, do not know whether peri‐infarction tight glycemic control or long‐term insulin therapy is the significant therapeutic intervention for the AMI population (Malmberg et al., 1995). It would seem of key importance to determine whether or not there is a therapeutic time window in cardiac patients with AMI for insulin therapy. Stricter and more rigorous measures should be implemented to impose early insulin administration as early as symptom onset in order to investigate whether or not there is a benefit of insulin on outcome in AMI. The most recent and encouraging IMMEDIATE trial, published in 2012, did find benefits of GIK administration in AMI patients with a 52% reduction in cardiac arrest or in‐hospital mortality with select group analysis even showing reduced infarct size on imaging (Selker et al., 2012).

## 4 GLYCEMIA IN CRITICALLY ILL PATIENTS WITH CS AND FUTURE PERSPECTIVES

### 4.1 Blood glucose targets in CS

Optimal blood glucose targets in CS are unknown. Recommendations for blood glucose management in MI patients complicated by CS are, therefore, unavailable and extrapolated from those applied in AMI and critically ill patients. Among CS patients' blood glucose targets of 8–10 mmol/L are recommended with the use of intravenous insulin (Capes et al., 2000; Finney et al., 2003; Thiele et al., 2019; Van de Werf et al., 2008; Van den Berghe et al., 2006). The reasoning supporting such practice stems from large studies that illustrate the U‐shaped relationship between glycemic controls and mortality in critically ill patients (Pinto et al., 2005; Siegelaar et al., 2010; The NICE‐SUGAR Study Investigators, 2009). Indeed, both episodes of hyperglycemia and hypoglycemia have a negative impact on survival in these patients (Pinto et al., 2005; Siegelaar et al., 2010; The NICE‐SUGAR Study Investigators, 2009). A randomized control trial conducted by Van den Berghe et al showed a clear survival benefit in critically ill surgical patients who were treated with intensive insulin therapy regardless of whether a previous diagnosis of diabetes had been made (Van den Berghe et al., 2001). The same results were not reproduced completely in medical ICU patients, although prolonged treatment with insulin for 72 h did show a survival benefit (Van den Berghe et al., 2006). Moreover, tight glycemic controls of 4.5 to 6.0 mmol/L with intensive insulin treatment as was protocoled in the NICE‐SUGAR trial showed an increase in mortality presumably due to episodes of hypoglycaemia (The NICE‐SUGAR Study Investigators, 2009). When analyzing the results of the major studies that investigate glucose targets with insulin administration in patients with acute coronary syndrome, DIGAMI, ECLA and GIPS showed decreased mortality with glucose targets between 6.93 and 10.9 mmol/L (Malmberg et al., 1995; The CREATE‐ECLA Trial Group Investigators*, 2005; Van der Horst et al., 2003). Having more permissive targets of less than 16 mmol/L as was the case of POL‐GIK trial increases mortality as does having a lower threshold of 3.96–10 mmol/L, in the HI‐5 study. Cardiac performance is directly impacted by both hyperglycemia and hypoglycemia, the latter being associated with an increased adrenergic tone that favors recurrent myocardial ischemia and the development of triggered ventricular arrhythmias (Chow et al., 2014; Zhang & Zhou, 2016).

### 4.2 Hyperglycemia and prognosis of ICU patients

Numerous adult and pediatric prospective and retrospective studies have established a positive correlation between hyperglycemia during critical illness and mortality, even at modest degrees of hyperglycemia (Macrae et al., 2014; Srinivasan, 2012; Van den Berghe et al., 2001; Vlasselaers et al., 2009). The development of hyperglycemia in the ICU setting is clearly correlated with increased duration of hospital stays, increased duration of mechanical ventilation, and increased likelihood of CPR and of developing infectious complications (Abdin et al., 2018; Dungan et al., 2009; Sung et al., 2005).

### 4.3 SGLT‐2 inhibitors and heart failure

In recent years the innovative SGLT‐2 inhibitors (iSGLT‐2s) have gained increasing popularity in the treatment of type II diabetes. Three large well‐powered RCT's, EMPAREG, DAPA‐HF, and EMPEROR showed major cardiovascular mortality benefits and decreased hospitalization rates for heart failure in the groups treated with iSGLT‐2 s (McMurray et al., 2019; Packer et al., 2020; Zinman et al., 2015). McMurray et al. (2019), the authors of DAPA‐HF, compared dapagliflozin, an SGLT2 inhibitor, to placebo among 4744 patients with HFrEF. Patients in this trial were adults of at least 18 years of age, with NYHA class II‐IV symptoms and an LVEF of 40% or less. The results showed a statistically significant reduction in rehospitalization for worsening HF, as well as a reduction in cardiovascular‐related deaths among patients with HFrEF, regardless of diabetes status (McMurray et al., 2019). The current mechanisms involved in the cardiovascular benefits of SGLT2 inhibitors remain a topic of active discussion. The authors cite the possible diuretic effect of dapagliflozin, as well as its potential effects on cardiac metabolism, fibrosis, and vascular function (McMurray et al., 2019). The positive effects of SGLT2 inhibitors may be mediated by the inhibition of sodium‐hydrogen exchange, but further studies are needed to elucidate the matter (Packer et al., 2017). These results are encouraging and further our understanding of the interaction between glucose metabolism and cardiac function. Interestingly, it would be of benefit to determine whether these new SGLT2 inhibitors have an impact on outcomes such as mortality and cardiac function in patients with HFpEF or in patients with AMI complicated by CS. If the theory that the diuretic properties of dapagliflozin are the cause of its positive cardiac effects, prospective analysis of urine output should show significantly increased 24‐h urine volume in the treated group. With all this in mind, it would seem that the interplay between glucose, inflammation, insulin, and cardiomyocytes warrants further research.

## 5 CONCLUSION

Several studies highlight the impact of glucose metabolism on cardiac function, with an emphasis on stress hyperglycemia and its cardiotoxic effects. Stress hyperglycemia and the myocardium intersect at a cardiometabolic carrefour where inflammation, oxidative stress, and reperfusion ischemia come together and interact. It would seem that the relationship between insulin therapy and heart function is complex and subtle, with the benefits of insulin therapy during stress hyperglycemia states in myocardial infarction still remaining controversial. Although a great deal of research has been undertaken to elucidate glucose‐heart interactions, there remains a lot of questions to be answered. The impact of stress hyperglycemia and its molecular pathogenesis on cardiac dysfunction, the optimal glucose targets as well as the insulin doses and timing to be recommended during states of acute heart failure and myocardial infarction are some of the topics and controversies that stimulate our curiosity. Moreover, with regard to which therapeutic intervention improves prognosis. There is no clear evidence available to distinguish between which of the two treatment strategies, being the achievement of a physiological state of euglycemia or the administrating insulin, improves survival in critically ill “cardiovascular” patients subject to stress hyperglycemia. Thinking ahead, the new SGLT2 inhibitors seem to provide a promising therapeutic strategy that targets the cardiometabolic interactions of glucose and the heart. Their impact on the outcome of acute heart failure as well as on HFpEF seems to be a promising area of research. In the future, it may also be of interest to focus scientific research on specific metabolomic profiles of cardiomyocytes during sates acute hyperglycemia, to further elucidate the impact of certain metabolic pathways on cardiac function. Perhaps the implementation of rigorous protocols of early insulin administration in cardiac ICU patients with stress hyperglycemia could shed light on the question of whether or not early administration of insulin during AMI alters prognosis. Because inflammation is the cornerstone of heart failure prognosis, and hyperglycemia is a poor prognostic indicator, in CS, it may also be time to investigate the impact of glycemia on CS prognosis in a prospective clinical trial. The molecular effects of an acute glucose insult on the cardiomyocyte, the pro‐inflammatory cascade that accompanies states of stress hyperglycemia, and the prognostic implications of hyperglycemia during heart failure remain a real challenge for cardiologists and intensivists.

## CONFLICT OF INTEREST

No conflicts of interest to declare.

## AUTHORS CONTRIBUTIONS

Each author contributed to the creating, editing, and finalizing of the article.
